# Alternating time spent on social interactions and solitude in healthy older adults

**DOI:** 10.1111/bjop.12586

**Published:** 2022-08-11

**Authors:** Minxia Luo, Theresa Pauly, Christina Röcke, Gizem Hülür

**Affiliations:** ^1^ University Research Priority Program (URPP) Dynamics of Healthy Aging University of Zurich Zurich Switzerland; ^2^ Department of Psychology University of Zurich Zurich Switzerland; ^3^ Center for Gerontology University of Zurich Zurich Switzerland; ^4^ Department of Psychology University of Bonn Bonn Germany

**Keywords:** event‐contingent ambulatory assessment, fatigue, homeostasis, life satisfaction, self‐regulation, social and solitary activities

## Abstract

Time spent on being with others (social interactions) and being alone (solitude) in day to day life might reflect older adults' agentic regulatory strategies to balance the needs to belong and to conserve energy. Motivated from a joint lifespan psychological and social relationship theoretical perspective, this study examined how time spent on social interactions and solitude alternatively unfolds within individuals in daily life, relating to individual differences in trait‐level well‐being and fatigue. Over 21 days, a total of 11,172 valid records of social interactions were collected from 118 older adults (aged 65–94 years) in a smartphone‐based event‐contingent ambulatory assessment study in Switzerland. On average, a social interaction episode lasted 39 min and a solitude episode lasted 5.03 hr. Multilevel models showed that, at the within‐person level, a longer‐than‐usual social interaction preceded and was followed by a longer‐than‐usual solitude episode. Moderator analyses showed that older adults with higher trait life satisfaction and lower trait fatigue spent even more time in social interactions after longer solitude episodes, amplifying the solitude‐then‐interaction association. Our findings suggest that whereas social interaction is a means to improve well‐being, solitude is also an integral part in older adults' daily life supporting energy recovery.

## BACKGROUND

Individuals' lifestyle is, at least in part, formed by personal choices of time and energy investment on different daily activities and is closely related to health and well‐being in older age (Horgas et al., 1998; Möwisch et al., 2022). The phase of older age in current Western culture, particularly postretirement, is considered to involve relatively few obligations and social expectations and is viewed as a time of leisurely and social pursuits that can be carried out and planned with fewer external restrictions (Freund, 2020). As such, how older adults regulate time spending in daily activities is an important topic for maintaining their health and well‐being.

Social interaction is recognized as an important daily activity for older adults' well‐being (Adams et al., 2011; Charles et al., 2021). However, recent studies have shown that benefits of social interactions on well‐being are subject to the law of diminishing returns and thus are not unlimited (Kushlev et al., 2018; Luo et al., 2022; Ren et al., 2021). These studies have raised an important question of how individuals should arrange their time in order to optimize their well‐being. Conversely, solitude, defined as the absence of social interaction (Burger, 1995; Larson, 1990), also serves multiple useful functions, such as preserving privacy and inner peace and replenishing (Coplan & Bowker, 2013; Long et al., 2003).

There has been little research examining how time is spent on social interactions and solitude sequentially in older adults' daily life. We propose that time spent with others (i.e. social interactions) and time spent alone (i.e. solitude) alternatively unfolding in daily life might reflect older adults' agentic regulatory processes to meet and balance the sense of belonging and the sense of energy. Further evidence is needed to form the basis for the development of guidance for lifestyle interventions for older adults. We draw from both social relationship and lifespan psychological theories and research to inform the current study.

### Social interactions as a means to well‐being and solitude as an opportunity to recharge

Several psychological theories have proposed that to belong and to be affiliated are fundamental psychological needs of human beings. For example, the belongingness hypothesis proposes that the need to belong is a pervasive drive that motivates human beings to form at least a minimum quantity and quality of interpersonal relationships and that failure to satisfy this fundamental need has a detrimental impact on well‐being (Baumeister & Leary, 1995). Similarly, the social affiliation model posits that individuals seek to maintain an optimal range of social contacts and that deviations from this equilibrium would motivate individuals to act and re‐establish the optimal range (O'Connor & Rosenblood, 1996). In line with these theoretical propositions, ample evidence has shown that engaging in social interactions promotes well‐being throughout adulthood and into old age, including state‐level positive affect, negative affect and life satisfaction (Sandstrom & Dunn, 2014; Sun et al., 2019).

On the other hand, the role of solitude has also been implicitly or explicitly acknowledged in theories on social interactions. Specifically, the belongingness hypothesis predicts that once a certain minimum quantity of social contacts has been achieved in a given time period, the motivation to further engage in social interactions should diminish (Baumeister & Leary, 1995). Accordingly, although implicit, the belongingness hypothesis suggests that individuals would return to solitude as a ‘default’ state once the minimum quantity is surpassed. Similarly, the social affiliation model posits that if excess contact is experienced, people will seek out solitude and that if too much solitude is experienced, they will again interact with others (O'Connor & Rosenblood, 1996). The authors referred to this homeostatic process as analogous to maintaining an optimal level of caloric intake, according to which individuals would eat or stop eating (O'Connor & Rosenblood, 1996). Different from the belongingness hypothesis, which only indirectly implies the role of solitude, this model explicitly states that both solitude and social interactions are essential phases in the homeostatic process of meeting the need to affiliate. Furthermore, the communicate bond belong theory posits a homeostatic model, wherein the motivation to socially engage and the motivation to conserve energy jointly determine whether an individual would engage in a social interaction (Hall & Davis, 2017). The theory proposes that individuals have a finite storage of energy at any given time to be spent on social interactions and that when this energy is expended, an individual will refrain from further interactions (Hall & Davis, 2017).

Taken together, social interactions and solitude are both important elements in a self‐regulatory process of meeting the need to belong and to conserve energy. In this process, social interaction is a means to fulfil the need to belong, whereas solitude is seen as a restorative process to renew energy for future social interactions (Baumeister & Leary, 1995; Hall & Davis, 2017). Thus, time spent on social interactions and solitude could be conceptualized as governed by the states of a sense of belonging or energy. In support of this view, research has shown that engagement in social interaction is associated with *concurrent* positive emotional states but *subsequent* states of fatigue (Leikas & Ilmarinen, 2017; Paolillo et al., 2018). Further, research has also shown that, at the state level, moments in solitude were not only associated with lower high‐arousal positive affect (e.g. happy, alert) and higher low‐arousal negative affect (e.g. sad, sleepy), but also were associated with higher low‐arousal positive affect (e.g. calm, quiet; Birditt et al., 2019; Pauly et al., 2017), which is reflective of relaxation (Vlemincx et al., 2015). While most studies have inquired about isolated effects of social interactions or solitude on well‐being (Birditt et al., 2019; Kushlev et al., 2018), examination of alternation of time spent across different activities could shed a new perspective. Some studies have shown that when individuals feel bad (vs. good) at the moment, they are more likely to engage in social interactions (vs. to work) in the next moment (Quoidbach et al., 2021; Taquet et al., 2016). As affective states are generated during a prior activity (social interaction vs. solitude), a prior activity could influence subsequent activity engagement.

**FIGURE 1:**
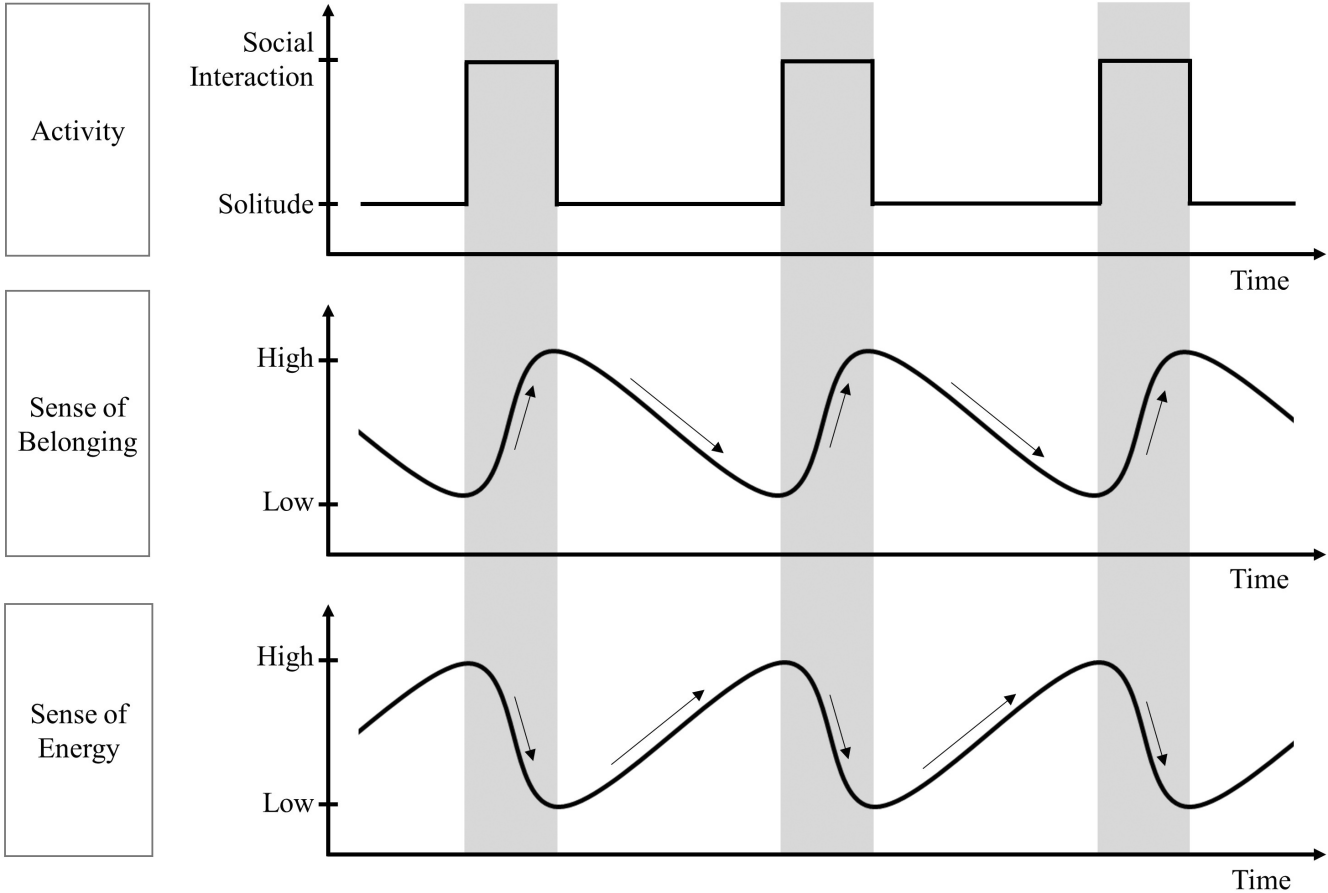
A conceptual figure of a self‐regulatory process to fulfil the need to belong and the need to conserve energy through alternating time spent on social interaction and solitude. Time spent with others (i.e. social interactions) and time spent alone (i.e. solitude) alternatively unfolds in daily life. Time spent on social interactions and solitude reflects state‐level variations in the sense of belonging and the sense of energy along the process. As individuals are expected to act as agents and regulate their need to belong and to conserve energy, time spent on one activity should be positively related to time spent on the preceding activity.

As illustrated in Figure 1, we propose that time spent on social interactions and solitude unfolding over time reflects individuals' state‐level sense of belonging and energy along the process. During social interactions, individuals consume their energy to fulfil their need to belong and, thus, the sense of belonging increases and the sense of energy reduces. During solitude episodes, individuals take a break from social interactions and have their energy recharged and, thus, the sense of energy increases but the sense of belonging reduces. Further, as individuals are expected to act as agents and regulate their need to belong and to conserve energy (Hall & Davis, 2017), we propose that time spent on one activity is influenced by time spent on the preceding activity.

### Self‐regulation of time spent on social interaction and solitude in older age

Alternation of social interactions and solitude could be particularly important for older adults, as maintaining emotional well‐being through social relationships becomes increasingly important in old age (Carstensen et al., 1999; Charles, 2010). Moreover, individuals actively shape their own development through adaptive strategies to bridge between desires and needs, existing resources, and contextual constraints, as outlined in the lifespan theory of selective optimization with compensation (Baltes & Carstensen, 1996). Faced with limited energy and future time perspective, older adults might strategically regulate their social relations to best serve their own well‐being (Lang, 2001). For example, research suggests that older adults keep contact with emotionally meaningful social ties and deliberately discontinue their relationships with weaker social ties (Lang & Carstensen, 1994; Lang et al., 2009). It has been shown that older adults with rich sensorimotor‐cognitive and social‐personality resources increased time spent with close social ties and also took more regenerative naps during the day, whereas older adults with poor personal resources reduced time spent with close social ties and also spent less time sleeping during daytime over a period of four years (Lang et al., 2002).

**FIGURE 2:**
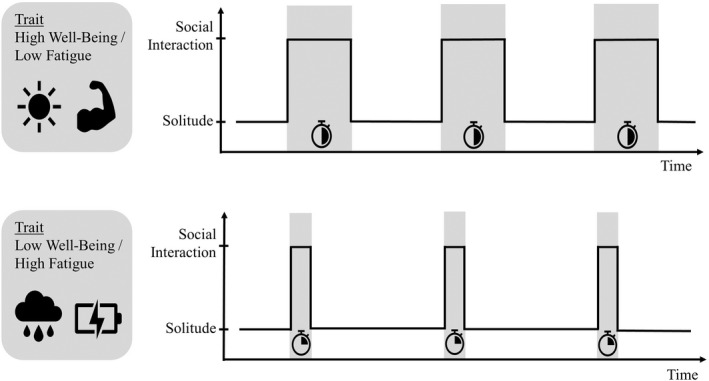
A conceptual figure on time spent on social interactions and solitude by individual differences in trait‐level well‐being and fatigue. Compared to older adults with lower trait well‐being and higher trait fatigue, older adults with higher trait well‐being and lower trait fatigue should experience even longer subsequent social interactions after longer prior solitude episodes. Moreover, older adults with higher trait well‐being and lower trait fatigue should experience subsequently shorter solitude episodes after longer prior social interactions.

Adopting lifespan psychological theoretical notions of resource allocation, individuals' self‐regulatory processes in alternating time spent on social interactions versus solitude may meaningfully differ among older adults as a function of their trait‐level well‐being and energy. As discussed above and shown in Figure 1, we propose that state‐level well‐being (achieved by meeting the need to belong) and energy are two factors that influence the process where older adults spend their time differently on social interactions and solitude (Baumeister & Leary, 1995; Hall & Davis, 2017; O'Connor & Rosenblood, 1996). To expand this idea further, we propose (as shown in Figure 2) that older adults with higher trait well‐being and lower trait fatigue might have more personal resources to be spent on social interactions. That is, they might overall have higher motivation and energy to engage in social interactions to fulfil the need to belong and require less time being alone to restore their energy before returning to social interactions.

We are not aware of any research to date that has examined the patterns of how time spending on social interactions and solitude alternately unfolds in daily life. In other words, little research has investigated whether and how prior interpersonal activity duration (i.e. social interaction or solitude) subsequently influences the other interpersonal activity duration (i.e. solitude or social interaction). Some studies have described time spent on lifestyle activities, including social and solitary activities, by the so‐called ‘yesterday interview’ (Lam & García‐Román, 2020; Lang et al., 2002). This method requires participants to list all activities and their duration on the prior day. Retrospective recall may introduce bias, particularly in older adults who experience memory decline (Hatt et al., 2021). Other studies used a signal‐contingent ambulatory assessment method, in which participants received prompts of questionnaires and reported the presence or absence of solitude or social interaction (Birditt et al., 2019; Kushlev et al., 2018). However, the studies with a signal‐contingent design could only approximate time spent on an activity as the proportion of that activity over all assessments and do not show how solitude and social interactions alternatively unfold in daily life.

### The current study

This study, for the first time, examined how solitude and social interactions alternatively unfold in older adults' daily life. According to lifespan developmental perspectives (Baltes & Carstensen, 1996; Lang & Heckhausen, 2006), we viewed older adults as adaptive agents and viewed time spent on daily activities as a result of older adults' agentic self‐regulation in resource allocation. We focused on older adults who were relatively healthy and community‐dwelling and assumed that participants had a similar level of agentic regulatory capacity.

We took a smartphone‐based event‐contingent study design to observe duration of social interaction and solitude episodes. We defined social interaction as having a conversation with someone in person, by telephone or digitally (Chan, 2018; Zhaoyang et al., 2018). We defined solitude as the absence of social interaction (Larson, 1990; Lay et al., 2020), which is distinct from aloneness characterized by the physical absence of other people (Lay et al., 2019). In this study, participants reported any social interaction and its time and duration as soon as it occurred. Duration of solitude was then specified as the duration of time when no social interactions were reported. The event‐contingent design overcomes memory biases of retrospective reports that can influence ‘yesterday interview’ results and offers better precision than signal‐contingent ambulatory assessment studies in estimating activity duration. Similar to Lam and García‐Román (2020) in their population survey on solitude time, we counted sleeping time as solitude episodes. In our conception, older adults could arrange their time spending according to the natural unfolding of their needs of belonging and energy conservation over time, without a specific constraint on time frame. For example, following several days of solitude, one might engage in longer social interaction than they typically do, so as to compensate for their lower (state) level of need to belong. Thus, in this study, a solitude episode could last up to several days and, in case of reporting no social interaction, even the whole study period.

We followed two research aims, which were motivated from a joint lifespan psychological and social relationship theoretical perspective. First, we aimed to examine within‐person associations between (1) prior solitude episode duration and subsequent social interaction duration; and (2) prior social interaction duration and subsequent solitude episode duration. As shown in Figure 1, we expected that individuals' prior solitude duration would positively predict subsequent social interaction duration and vice versa. Specifically, we expected that a longer solitude episode goes along with higher sense of energy but lower sense of belonging and, thus, it triggers a higher likelihood (motivation) to engage in longer social interactions subsequently. In reverse, we expected that a longer social interaction episode goes along with higher sense of belonging but lower sense of energy and, thus it gives rise to a stronger likelihood (motivation) to engage in longer solitude subsequently. Note that we did not expect a positive feedback loop here that episodes of social interaction and solitude would become longer and longer over time. We propose that alternation of time spending on social interactions and solitude reflect the states of motivation to meet the need to belong and to restore energy. Yet, we also acknowledge that time spending on daily activities is not only initiated by our participants according to their needs. After all, time spent on social interactions can also be shaped by environmental opportunities (e.g. availability of social ties, interactions initiated by the others) or health status (Cornwell & Laumann, 2015, 2018). There is also a certain degree of randomness in daily life.

Second, we aimed to examine whether and how the within‐person processes differed between older adults with different trait‐level well‐being and energy. We expected older adults with more personal resources (indicated by high trait‐level well‐being and low trait‐level fatigue) would strategically spend more time in social interactions (for their need to belong/well‐being) and less time in solitude episodes (for energy conservation). As shown in Figure 2, compared to those who had lower trait well‐being and higher trait fatigue, we expected that older adults with higher trait well‐being and lower trait fatigue would experience overall longer social interactions and even longer subsequent social interactions after longer prior solitude episodes. Further, we also expected older adults with higher trait well‐being and lower trait fatigue to experience shorter solitude episodes and even shorter solitude episodes after longer prior social interactions.

## METHODS

This study aimed to examine alternation patterns of social interactions and solitude in healthy and community‐dwelling older adults. To do so, we use data from a larger project on digitalization and social lives of older adults (Macdonald & Hülür 2020), which included a 21‐day event‐contingent data collection in older adults aged 65 years and above in Switzerland. The study procedures were conducted according to the Declaration of Helsinki and were approved by the Ethics Committee of the Faculty of Arts and Social Sciences at the University of Zurich (Nr. 19.2.17).

### Participants

A total of 120 older adults from German‐speaking regions of Switzerland participated in the study. Based on prior research on daily social interactions (Ram et al., 2014; Wood et al., 2018), we expected seven social interactions per day and conducted power analyses using Monte Carlo simulations in MPlus (Muthén & Muthén, 2017) to determine the sample size. The sample size of 120 participants was shown to be sufficient to detect small (i.e. 0.2 *SD* units) intraindividual effects and cross‐level moderation effects with a power of β = .80. Participants were recruited via advertisements in local and national newspapers and through a database of participants hosted at the University of Zurich. Participants had to meet the following criteria: Using digital devices to communicate, having sufficient hearing and vision, being fluent in German, and being 65 years or older. Participants were compensated with 150 Swiss Francs for their participation.

### Study design and procedures

After providing informed consent, participants took part in a baseline session where they received detailed instructions on the study protocol and a take‐home questionnaire to collect their demographic information and psychological variables, including positive and negative affect, life satisfaction and fatigue. After the baseline session, participants were given an iPhone 4S to complete a 21‐day event‐contingent observation period. The questionnaires were administered with the app ‘iDialogPad’ (G Mutz). Participants were asked to record any spoken interactions (i.e. face‐to‐face, telephone, video chat) that lasted longer than 5 min and any text‐based conversations (i.e. text message, email, letter, social media). The five min cut‐off was implemented on the basis of earlier research on meaningful social interactions in daily life to reduce participant burden during the data collection period (Reis & Wheeler, 1991). More details on study procedures have been documented in (Macdonald & Hülür 2020). The compliance rate was high at 90%, with participants completing an average of 18.96 (*SD* = 2.77) out of 21 possible days. Some participants completed interaction reports beyond the 21‐day period. Number of social interactions reported per day decreased over the study period. We excluded data from two participants: One participant did not complete the take‐home questionnaire and the other one misunderstood the instructions on recording social interactions.

### Measures

#### Social interaction duration

Participants reported the duration of each spoken social interaction (i.e. face‐to‐face, telephone, video chat) by answering the following question: ‘How long did the conversation last?’ (German ‘Wie lange hat das Gespräch gedauert?’). Conversations with the same person that happened intermittently within the same context, not necessarily engaging in a lengthy discussion but just exchanging a few words now and then, were counted as one social interaction. For example, watching a movie together over 2 hr was counted as one social interaction, because they may have chatted from time to time during the movie. We removed 265 (0.02%) interactions that were reported as shorter than 5 min as they did not meet the inclusion criteria. Different from spoken interactions, participants did not report the duration of text‐based conversations (i.e. text message, email, letter, social media). Intermittent text‐based conversations that extend over hours often include other ongoing activities. We counted each text‐based conversation (36% out of all reported conversations) as 5 min. Changing the duration to 10 min did not impact our findings.

#### Solitude duration

Based on information on social interaction onset and duration, we calculated the time in solitude when no social interactions were reported. Some social interactions overlapped in time and the solitude episodes in between were smaller than zero. For example, a text message could be sent during a telephone call. A total of 719 records (0.06%) of these negative solitude episodes were recoded as zero, indicating that there was no interval between two overlapping social interactions. A follow‐up analysis excluding these records led to identical findings. Additionally, sleeping time was counted in the solitude episodes. In our study, there were 2403 (22%) solitude episodes that lasted overnight from a previous day to the next day(s). The average duration of these episodes was 16.94 hr (*SD* = 10.47 hr, range = 0.43–159.83 hr [6.7 days]).

#### Trait positive and negative affect

The German version of The Positive And Negative Affect Schedule (Breyer & Bluemke, 2016; Watson et al., 1988) was used to examine participants' trait positive affect and negative affect. Each of the two trait affective factors contains 10 emotions. For example, *positive affect* includes ‘excited’ and ‘inspired’ and *negative affect* includes ‘upset’ and ‘nervous’. On a scale from 1 (very slightly or not at all) to 5 (extremely), participants indicated their answer to the question ‘how often have you felt this feeling during the last year’. Scores were averaged for each affective factor. Higher scores indicated higher affect levels in each domain. Cronbach's alpha was 0.80 for trait positive affect and 0.84 for trait negative affect.

#### Trait life satisfaction

The German version of the Satisfaction With Life Scale (Diener et al., 1985; Janke & Glöckner‐Rist, 2014) was used to evaluate participants' trait life satisfaction. Participants indicated the extent to which they agreed or disagreed with five statements (e.g. ‘In most ways my life is close to my ideal’) on a 7‐point scale (1 = strongly disagree and 7 = strongly agree). Higher scores indicated higher life satisfaction. Cronbach's alpha for trait life satisfaction was 0.90.

#### Trait fatigue

The 36‐Item Short Form Health Survey (SF‐36; Ware Jr. & Sherbourne, 1992) yields an ‘energy/fatigue’ subscale score based on four items (e.g. ‘Did you feel worn out?’). The German version of this subscale (Bullinger & Kirchberger, 1998) was used to indicate participants' trait fatigue level. Scores range from 0 to 100, with higher scores suggesting more fatigue. Cronbach's alpha for trait fatigue was 0.78.

#### Covariates

We controlled for covariates that have been found to be closely related to social interactions and well‐being (Adams et al., 2011; Charles et al., 2021). Covariates included participants' *age* in years, *gender* (0 = women, 1 = men), number of physician‐diagnosed *health conditions* during the last 2 years, and *marital status* (1 = married, 0 = not married). We also controlled for *the number of days of the study* (ranging from 0 to the total number of days reported) and a binary variable indicating weekday (= 0) versus weekend (= 1).

### Analytical approach

Multilevel models were used to examine the alternation of time spending on the two activities. Alternation is operationalized as the associations between prior activity duration (i.e. social interaction or solitude) and subsequent activity duration (i.e. solitude or social interaction). According to procedures that are typically used to analyse ambulatory assessment data (Bolger & Laurenceau, 2013), we split the time‐varying predictor variables into between‐ and within‐person components. The between‐person component of prior activity duration refers to average duration of an activity (social interaction or solitude) of a participant over the study observation period. The within‐person component refers to the deviation of that prior activity duration from the person‐specific average activity duration (i.e. the between‐person component). For example, assume a participant typically spends 0.5 hr in social interactions. A score of 1.5 would be separated into a between‐person component of 0.5 hr and a within‐person component of 1 hr, meaning that the participant spent 1 hr longer than usual in this particular social interaction episode. In the first step, we examined the associations at the within‐person level. The models were specified as

(1) $${\text{Outcome}}_{\mathrm{ti}}=\beta_{0i}+\beta_{1i}\;\left(\text{prior within}-\text{person}\;{\text{activity duration}}_{\mathrm{ti}}\right)+e_{\mathrm{ti}}$$

(2) $$\beta_{0i}=\gamma_{00}+u_{0i};$$

(3) $$\beta_{1i}=\gamma_{10}+u_{1i};$$

where Outcome_*ti*_, person *i*'s activity duration at measurement point *t*, is a function of: β_0*i*_, a person‐specific intercept parameter representing the individual mean level in the outcome contingent on the other model predictors, β_1*i*_, which captures the within‐person effect of the prior activity duration, and *e*_*ti*_ represents the residual within‐person variance. In addition, γ_00_ represents the sample average of the outcome; *u*_0*i*_ is the deviation of a participant's outcome score from the sample‐average score; γ_10_ represents the sample‐level association (slope) of prior within‐person activity duration on the outcome; and *u*_1*i*_ is the deviation of a participants' slope from the sample‐level slope.

In the second step, we examined effects of between‐person moderators on the outcome and the within‐person associations. That is, we kept the Level 1 Equation (1) as it is and added moderators to the Level 2 Equations (2) and (3), turning them into Equations (4) and (5) as follows:

(4) $$\beta_{0i}=\gamma_{00}+\gamma_{01}\;\left({\text{moderator}}_{i}\right)+u_{0i};$$

(5) $$\beta_{1i}=\gamma_{10}+\gamma_{11}\;\left({\text{moderator}}_{i}\right)+u_{1i};$$

where γ_01_ indicates the main effect of the moderator on the outcome variable and γ_11_ indicates a cross‐level interaction (the strength of the association between the Level 1 predictor and the outcome depends on the moderator). We first examined models without any covariates and then added the covariates as main effects into the models. We standardized all moderators with a Likert scale (i.e. trait positive and negative affect, trait life satisfaction, trait fatigue) into *z*‐scores (*M* = 0; *SD* = 1) for ease of interpretation and centred the covariates of age, gender, health conditions and marital status at the sample level. Analyses were conducted in R (R Core Team, 2013) using the ‘nlme’ package (version 3.1‐149; Pinheiro et al., 2017). We calculated pseudo R‐Squared of the models for explained variance with the ‘MuMIn’ package (version 1.43.17; Barton & Barton, 2015). To determine effect sizes of the key findings, we also calculated standardized regression estimates (STEs; Hülür et al., 2014). Specifically, we obtained the standard deviation of the within‐person and between‐person variances in multilevel models and then used the respective standard deviation to rescale the unstandardized regression estimates, in order to represent effect sizes in between‐person or within‐person standard deviation units. Statistical significance was evaluated at *p* < .05.

## RESULTS

We examined a total of 11,172 valid records of social interactions. The 118 participants had a mean age of 72 years (*SD*_age_ = 5 years, range_age_ = 65–94 years; 40% women). Most participants (97%) were retired and 55% of them were married. After secondary school education (36% participants received a degree qualifying for university education), the majority of participants (98%) completed further training and about 22% completed university education. About 52% participants lived in cities, 16% in small towns and 33% in villages and small villages. About 18% of participants had an income up to 4′000 Fr., 53% of them had an income between 4′001 to 8′000 Fr., and 22% of them had an income of more than 8′000 Fr per month (5% participants did not report their income).

On average, a social interaction episode lasted 0.65 hr (i.e. 39 min, *SD* = 1.36, Mdn = 0.17, range = 0.08–17.5) and a solitude episode lasted 5.03 hr (*SD* = 8.14, Mdn = 1.70, range = 0–159.83 [i.e. 6.7 days]). On a 1–5 scale, participants reported an average trait positive affect of 3.82 (*SD* = 0.51) and an average trait negative affect of 1.62 (*SD* = 0.52). They reported an average score of 5.39 (*SD* = 1.12) in trait life satisfaction on a 1–7 scale and 29.41 (*SD* = 14.13) in fatigue on a 0–100 scale. Participants had on average 2.30 (*SD* = 1.93) health conditions.

**FIGURE 3:**
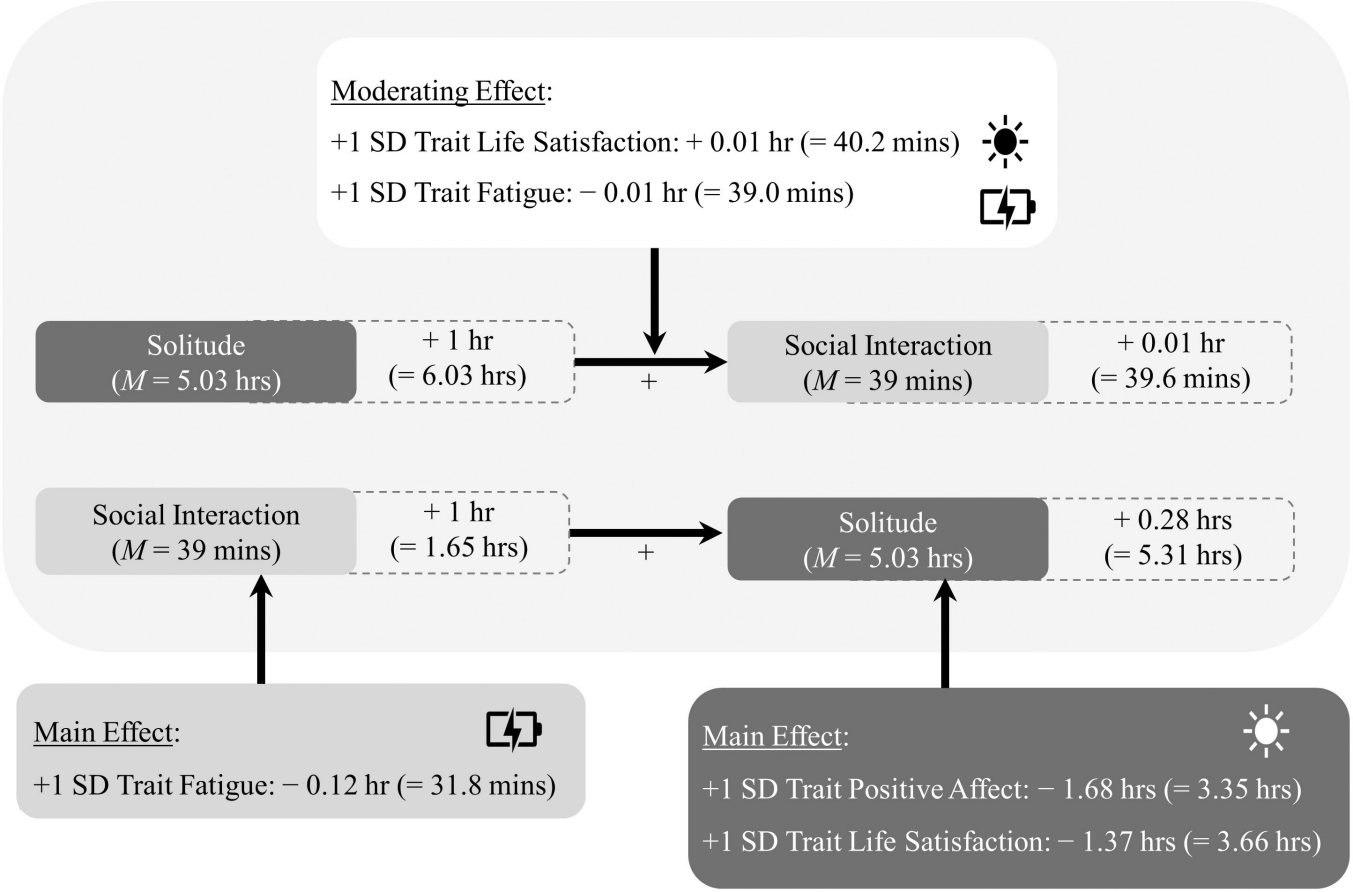
Overview of key findings: Alternating time spent on social interactions and solitude in older adults with different trait levels of well‐being and fatigue. For an older adult with a typical (sample‐average) 0.65 hr (i.e. 39 min) social interaction and a typical (sample‐average) 5.03 hr solitude episode, a 1 hr longer‐than‐usual solitude episode (i.e. 6.03 hr) preceded a subsequent 0.01 hr (i.e. 0.6 min) longer‐than‐usual social interaction (i.e. 39.6 min). In reverse, a 1 hr longer‐than‐usual social interaction (i.e. 1.65 hr) preceded a 0.28 hr (i.e. 16.8 min) longer‐than‐usual solitude episode (i.e. 5.31 hr). Further, compared to participants with a sample‐average level of trait life satisfaction and trait fatigue, after a 1 hr longer‐than‐usual solitude episode (i.e. 6.03 hr), participants with 1 *SD* higher trait life satisfaction experienced a 0.01 hr (i.e. 0.6‐min) even longer social interaction (i.e. 40.2 min) and participants with 1 *SD* higher trait fatigue experienced a 0.01 hr (i.e. 0.6‐min) shorter social interaction (i.e. 39 min). Regarding main effects, longer solitude duration was associated with lower trait positive affect and lower trait life satisfaction, whereas longer social interaction duration was associated with lower trait fatigue.

Table 1 shows the results for the first research question that focused on the within‐person associations. Specifically, a prior longer solitude episode was associated with a subsequent longer social interaction (γ_10_ = 0.01, SE = 0.003, *p* < .001, STE = 0.08). That is, when the prior solitude episode was 1 hr longer than usual, participants subsequently engaged in a 0.01 hr (i.e. 0.6 min) longer‐than‐usual social interaction. In reverse, when the prior social interaction was 1 hr longer than usual, participants subsequently experienced a 0.28 hr (i.e. 16.8‐min) longer solitude episode (γ_10_ = 0.28, SE = 0.08, *p* < .001, STE = 0.05). Please refer to Figure 3 for a graphical description of these findings. When adding covariates to the model (see Table S1), longer solitude duration was associated with older age and weekend days. Longer social interaction duration was associated with longer time in the study (i.e. higher number of days) and being at the weekend. Adding covariates into the models yielded similar findings.

**TABLE 1:** Effects of prior activity duration on subsequent the other activity duration

Parameter	Subsequent solitude duration			Subsequent social interaction duration		
	Est.	SE	STE	Est.	SE	STE
Fixed effects						
Intercept (γ_00_)	7.85*	0.63	1.40	0.76*	0.05	4.26
Prior activity duration (social interaction/solitude) (γ_10_)	0.28*	0.08	0.05	0.01*	0.003	0.08
Random effects						
*SD* (intercept, *u*_0*i*_)	6.82			0.57		
*SD* (slope, *u*_1*i*_)	0.43			0.03		
Corr (intercept‐slope)	−0.20			0.77		
*SD* (residual, *e*_*ti*_)	7.16			1.26		
Pseudo *R* ^2^	18.8%			15.0%		

Abbreviations: Corr, correlation; Est., estimate; *SD*, standard deviation; SE, standard error; STE, standardized regression estimate.

* *p* < .05.

Results of the second research aim on between‐person differences in the within‐person associations are shown in Tables 2 and 3. As shown in Table 2, lower trait positive affect (γ_01_ = −1.68, SE = 0.62, *p* = .008, STE = −0.24) and lower trait life satisfaction (γ_01_ = −1.37, SE = 0.63, *p* = .030, STE = −0.18) were associated with overall higher solitude duration (i.e. main effects), while trait negative affect and trait fatigue were unrelated to overall solitude duration. None of the trait well‐being and fatigue variables moderated the within‐person interaction‐then‐solitude association. As shown in Table 3, higher trait fatigue (γ_01_ = −0.12, SE = 0.05, *p* = .033, STE = −0.20) was related to lower overall social interaction duration (i.e. main effect), while trait positive and negative affect and trait life satisfaction were unrelated to overall social interaction duration. Furthermore, trait life satisfaction (γ_11_ = 0.01, SE = 0.003, *p* = .019, STE = 0.04) and trait fatigue (γ_11_ = −0.01, SE = 0.003, *p* = .032, STE = −0.04) moderated the within‐person solitude‐then‐interaction association. That is, compared to participants with a sample‐average trait life satisfaction and trait fatigue, after a 1 hr longer‐than‐usual solitude episode, older adults with 1 *SD* higher trait life satisfaction experienced a 0.01‐hr (γ_11_) even longer social interaction and older adults with 1 *SD* higher trait fatigue experienced a 0.01‐hr (γ_11_) shorter social interaction. Please refer to Figure 3 for a graphical description of these findings. Adding covariates to the models yielded similar findings, as shown in Tables S2 and S3.

**TABLE 2:** Effects of prior social interactions duration on subsequent solitude duration moderated by trait‐level well‐being and fatigue

Parameter	Outcome: Subsequent solitude duration											
	Moderator: positive affect			Moderator: negative affect			Moderator: life satisfaction			Moderator: fatigue		
	Est.	SE	STE	Est.	SE	STE	Est.	SE	STE	Est.	SE	STE
Fixed effects												
Intercept (γ_00_)	7.85*	0.62	1.40	7.85*	0.64	1.40	7.85*	0.62	1.40	7.85*	0.64	1.40
Prior social interaction duration (γ_10_)	0.29*	0.08	0.05	0.29*	0.08	0.05	0.29*	0.08	0.05	0.29*	0.08	0.05
Moderator (γ_01_)	−1.68*	0.62	−0.24	0.42	0.64	0.06	−1.37*	0.63	−0.18	0.05	0.64	0.01
Prior social interaction duration × moderator (γ_11_)	−0.09	0.09	−0.02	0.07	0.08	0.01	−0.10	0.08	−0.02	0.07	0.08	0.01
Random effects												
*SD* (intercept, *u*_0*i*_)	6.65			6.8			6.71			6.86		
*SD* (slope, *u*_1*i*_)	0.47			0.43			0.43			0.42		
Corr (intercept‐slope)	−0.26			−0.21			−0.27			−0.18		
*SD* (residual, *e*_*ti*_)	7.16			7.16			7.16			7.16		
Pseudo *R* ^2^	18.9%			18.8%			18.9%			18.8%		

Abbreviations: Corr, correlation; Est., estimate; *SD*, standard deviation; SE, standard error; STE, standardized regression estimate.

* *p* < .05.

**TABLE 3:** Effects of prior solitude duration on subsequent social interaction duration moderated by trait‐level well‐being and fatigue

Parameter	Outcome: Subsequent social interaction duration											
	Moderator: positive affect			Moderator: negative affect			Moderator: life satisfaction			Moderator: fatigue		
	Est.	SE	STE	Est.	SE	STE	Est.	SE	STE	Est.	SE	STE
Fixed effects												
Intercept (γ_00_)	0.76*	0.05	4.26	0.76*	0.05	4.26	0.76*	0.05	4.26	0.76*	0.05	4.26
Prior solitude duration (γ_10_)	0.01*	0.003	0.08	0.01*	0.003	0.08	0.01*	0.003	0.08	0.01*	0.003	0.08
Moderator (γ_01_)	0.03	0.05	0.06	−0.10	0.05	−0.19	0.07	0.05	0.11	−0.12*	0.05	−0.20
Prior solitude duration × Moderator (γ_11_)	0.004	0.003	0.02	−0.002	0.003	−0.01	0.01*	0.003	0.04	−0.01*	0.003	−0.04
Random effects												
*SD* (intercept, *u*_0*i*_)	0.57			0.56			0.56			0.56		
*SD* (slope, *u*_1i_)	0.03			0.03			0.03			0.03		
Corr (intercept‐slope)	0.77			0.77			0.77			0.76		
*SD* (residual, *e*_*ti*_)	1.26			1.26			1.26			1.26		
Pseudo *R* ^2^	15.0%			15.0%			15.1%			15.1%		

Abbreviations: Corr, correlation; Est., estimate; *SD*, standard deviation; SE, standard error; STE, standardized regression estimate.

* *p* < .05.

## DISCUSSION

This study examined alternation of time spent on social interactions and solitude in older adults as a reflection of resource allocation in the social domain in later life (Baltes & Carstensen, 1996). We hypothesized on the basis of several psychological theories from the social relations domain (Baumeister & Leary, 1995; Hall & Davis, 2017; O'Connor & Rosenblood, 1996), that time spent on social interactions and time spent on solitude were associated with each other. We further hypothesized that this process differed between people with divergent resources, that is trait‐level well‐being and energy. Results showed that longer‐than‐usual solitude episodes were followed by longer‐than‐usual social interactions and vice versa. Furthermore, these within‐person processes were in part moderated by older adults' trait life satisfaction and trait fatigue (only for the solitude‐then‐interaction association).

### Alternating time spent on social interactions and solitude in healthy older adults

At the within‐person level, our findings show that a longer‐than‐usual solitude episode preceded and was followed by a longer‐than‐usual social interaction. Notably, the effect sizes (STE) for both associations were comparable, although the unstandardized coefficients (duration in minutes) were different (interaction‐then‐solitude association: STE = 0.05, *b* = 0.28; solitude‐then‐interaction association: STE = 0.08, *b* = 0.01; see Table 1). This might be due to the fact that the average duration and the standard deviation of solitude and social interaction episodes were quite different (solitude: *M* = 5.03 hr, *SD* = 8.14 hr; social interaction: *M* = 0.65 hr, *SD* = 1.36 hr). The similar effect sizes suggest that compared to the own typical time in social interaction and in solitude, for each individual, the time change from social interaction to solitude was of similar size to the relative time change from solitude to social interaction. In line with our expectation, duration of solitude and social interactions mutually and positively interacted with each other within individuals. In other words, time spent on one activity is related to time spent on the preceding activity. We interpret that these positive within‐person associations reflect an agentic regulatory process involved in how time is spent in old age. As shown in Figure 1, time spent on social interactions and solitude episodes is invested to fulfil and balance the state‐level needs to belong and to restore energy. Longer time in social interactions may have better facilitated fulfilment of the state‐level need to belong, consuming more state‐level energy. Once the state‐level need to belong is satiated and the state‐level energy is depleted, a longer solitude episode follows. During a longer solitude episode, the state‐level need to belong becomes deprived and state‐level energy gets sufficiently recharged, inducing a subsequent longer social interaction.

Our findings are in line with theories on social interactions that emphasize the role of self‐regulatory processes where social interactions and solitude alternately unfold over time, such as the belongingness hypothesis (i.e. the motivation to engage in social interactions is subject to the law of diminishing returns, such that solitude is implied to exist between social interactions; Baumeister & Leary, 1995), the social affiliation model (i.e. social interactions and solitude are alternatively sought out or paused for maintenance of an optimal level of affiliation; O'Connor & Rosenblood, 1996) and the communicate bond belong theory (i.e. the motivation to belong and motivation to conserve energy jointly drive the selection of engagement in social interactions and solitude; Hall & Davis, 2017). Relatedly, two prior studies on social interactions and motivation have shown that desire to be alone or to be with others at a previous moment was associated with the corresponding activity measured in the next moment (Hall, 2017; O'Connor & Rosenblood, 1996). Findings of these studies suggest that current activities are undertaken in accordance with prior desires. Our findings extend the past studies by showing that longer time spent on one activity led to longer time spent on the other activity. According to our conception, time spent on activities might have reflected state‐level motivation to belong and/or to conserve energy.

The perspective to view social interactions as a means to well‐being is in line with ample existing evidence on the positive impact of social interactions and the negative impact of loneliness on health and well‐being (Adams et al., 2011; Charles et al., 2021; Ong et al., 2016). Beyond that, our findings show that time spent on a prior activity might have an effect on time spent on a subsequent activity. This suggest that not just social interaction but also solitude might be an integral and important part of older adults' daily life (Coplan et al., 2019), as solitude provides an opportunity for recovery from energy depletion associated with social interactions (Hoppmann et al., 2021). Research shows that solitude is functional as an adaptive emotional regulatory means to help individuals return to quiet or calm affect after an excitement or angry episode (Nguyen et al., 2018). Moreover, being in solitude due to autonomous motivation is associated with personal well‐being (Lay et al., 2020; Nguyen et al., 2019). On the other hand, engaging in daily social activities consumes energy and motivation (Cardini & Freund, 2019, 2020; Huxhold et al., 2022). In turn, our findings highlight the importance to critically examine social interactions and solitude in relation to state‐level well‐being and energy recovery, which could improve our understanding of time orchestration in older adults' daily activities.

### Effects of trait well‐being and trait fatigue on time spent on social interactions

Our findings at the between‐person level are also in line with our expectations. Regarding main effects, overall higher solitude duration was associated with lower trait positive affect and lower trait life satisfaction, whereas higher overall social interaction duration was associated with lower trait fatigue (Figure 3). These findings are in line with previous evidence that trait well‐being is associated with more time in social interactions (Adams et al., 2011; Milek et al., 2018) and trait fatigue is associated with fewer physical and mobility activities (Pérez et al., 2021), although we did not find any significant associations between average length of social interactions or solitude and positive and negative affect. One reason might be that most studies have quantified social interactions by its frequency (Adams et al., 2011), but this study quantified social interactions by its duration. There might be differences between frequency and duration of social interactions in relation to positive and negative affect, which, in turn, further highlight the necessity of examining duration of time spent on social interactions to understand predictors and correlates of older adults' well‐being.

As shown in Figure 3, compared to participants who had a sample‐average trait‐level life satisfaction and fatigue, older adults with higher trait life satisfaction and lower trait fatigue level experienced an even longer social interaction after a longer solitude episode. Older adults with higher trait‐level well‐being and lower trait‐level fatigue might be those who are more effective in using social interactions as a strategy to fulfil the need to belong and require less restorative breaks in solitude. Older adults are viewed as adaptive agents to coordinate their time spent on different activities according to their desires, well‐being and energy (Hall & Davis, 2017). These findings might reflect that older adults with different personal resources in well‐being and energy could afford to have different patterns of time spent on social interactions and solitude (Baltes & Lang, 1997; Lang et al., 2002). Furthermore, our findings are in line with research on personality and everyday social behaviours. Specifically, extraversion, which is often measured with items also capturing facets of well‐being (e.g. positive emotionality) and vigour (e.g. being energetic; Fleeson, 2001) has been linked to more and longer social interactions (Nezlek et al., 2011).

The moderating effects reflected a view that personal resources are the antecedents of time spending on different activities, in line with lifespan psychological theories (e.g. Baltes & Lang, 1997). In contrast, it is also conceivable that trait‐level well‐being and fatigue could be outcomes, rather than moderators, in line with our conception of the within‐person process (Figure 1). That is, the behavioural patterns of engaging in longer social interactions and in longer social interactions after longer solitude episodes may also lead to higher state‐ and (over time) higher trait levels of well‐being and lower fatigue. This conception of a reciprocal relation over time is also in line with the broaden‐and‐build theory, which posits that positive emotions broaden an individual's momentary thought‐action repertoire, which in turn builds the individual's personal resources (Fredrickson, 2001). Nevertheless, to further understand state‐ and trait levels of well‐being and fatigue in relation to social interactions and solitude would require future studies that combine data on short‐ and long‐term time scales.

### Alternation of time spent on social and solitary activities: a complex phenomenon?

We did not find any significant moderating effects of trait‐level well‐being and fatigue on the within‐person interaction‐then‐solitude association (only on the reverse within‐person solitude‐then interaction association). Although trait‐level well‐being and fatigue might explain time spent on social interactions conditional on prior solitude episodes, they did not seem to have an effect on time spent on solitude conditional on prior social interactions. To understand the results, we offer the following perspectives.

First, in line with our conception of a regulatory process consisting of social interactions as a means to well‐being and solitude as an opportunity to take a break and recharge, our findings may have suggested that engagement in social interactions differed between older adults with different available resources, but everybody might have returned to solitude of similar duration as a default state after a social interaction. More specifically, the transition from solitude to social interaction might need motivation and energy to engage in (e.g. setting up to go for a walk with a friend) and thus individuals with lower trait levels of well‐being and fatigue might have difficulties to engage in social interactions. In contrast, the transition from social interaction to solitude might require less energy and be more under older adults' control (e.g. leaving a conversation) and, thus, there may be less room for between‐person differences to be moderated by our target variables. Additionally, the association between solitude duration and energy recovery might not be linear. Thus, there might be a threshold when energy levels are restored and there is no benefit of additional solitary time. Thus, individual differences in trait‐level well‐being and fatigue do not necessarily need to have an association with time spent on solitude.

Second, solitude is a complex phenomenon (Coplan & Bowker, 2013), which may not only be determined by personal resources in well‐being and fatigue, but also other contextual (e.g. doing what, being where, motivation) and individual factors (e.g. mental health, social relations). For example, on days when young and older adults had more time to themselves, they felt less lonely if they were more creative than usual (Pauly et al., 2021). Being alone while walking in a municipal park was more restorative than walking with others, but walking with others along city streets was more relaxing than walking alone (Johansson et al., 2011). Solitude could be experienced out of external constrains, for example a lack of social company, but could also be experienced because of intrinsic motivation to be alone (Lay et al., 2020; Thomas & Azmitia, 2019). Additionally, solitude might be detrimental for depressive patients or adults with the tendency to ruminate over negative thoughts, but it could be good for individuals who have high social self‐efficacy with respect to social skills or have a trait‐like tendency of preferring to be alone (Coplan et al., 2019; Lay et al., 2019). Time in solitude might be good for well‐being in adults with conflicting or low‐quality social relationships (Birditt et al., 2019; Pauly et al., 2018). Thus, given its complex nature, solitude may have different implication for individuals with different trait‐level well‐being and fatigue, and this might have been reflected in the non‐significant moderating effects.

Similarly, research has shown that associations between social interactions, well‐being and fatigue are also complicated. For example, associations of social interactions with well‐being and health could differ according to subjective evaluations of interaction quality (Sun et al., 2019; Zhaoyang et al., 2019) and interaction modalities (Macdonald et al., 2021; Nguyen et al., 2021). Moreover, associations between the frequency and duration of social interactions and well‐being are shown to be curvilinear and differ between older adults with different assumed levels of satiation for the need to belong (Luo et al., 2022). In turn, a 1‐hr increase in social interactions might carry different weight for older adults with different typical amounts of social interaction. Additionally, research has shown that at the within‐person level, moments of social interaction are related to concurrently more positive state mood and lower state fatigue, but to subsequent higher state fatigue shortly afterwards (Leikas & Ilmarinen, 2017; Pickett et al., 2020). These findings speak to a hypothesis that although social interactions might go along with enhanced feelings of energy throughout the activity, they might deplete (mental or physical) resources, whose effects manifest in a delayed fashion. Thus, our sole focus on time duration, as one dimension of the quantitative aspect of activities, may have led us to overlook the complexity of time spent in social interactions and solitude and their associations with well‐being and fatigue. It is beyond the scope of this paper to examine all these factors, whose effects should be examined in detail in future studies.

Relatedly, some studies viewed state‐level affective well‐being as a signal that helps individuals prioritize their goals and motivation and coordinate daily activities (Carver & Scheier, 2013; Clore & Huntsinger, 2007). In line with this perspective, some studies have shown that momentary affective well‐being (e.g. feeling good vs. bad) could influence the next moment's activity engagement (e.g. social interaction vs. activities that promote long‐term payoff; Elmer, 2020; Quoidbach et al., 2019; Riediger & Luong, 2016). As affective states could serve as a feedback control signalling whether one's desires are being met, we reason that affective states might have reflected variations in motivation for fulfilling the need to belong and energy conservation. In this case, a self‐regulatory process may function as follows: Time spent on social interactions and solitude is an observable behaviour, driven by the motivation to belong and the motivation to conserve energy, wherein affective states provide information signalling the necessity for a transition.

Finally, we acknowledge several limitations of our research. First, self‐reported social interaction duration might be subject to the influence of social desirability, cognitive biases and cultural norms (Scollon et al., 2009). Participants might have estimated more or less time in social interactions depending on the quality or significance of those interactions. This is especially relevant for our sample of older adults, who prioritize positive emotional experiences according to the socioemotional selectivity theory (Carstensen et al., 1999). Relatedly, we might have overlooked brief spoken interactions that were shorter than 5 min (e.g. greeting neighbours or postal worker) and included them in solitude episodes. Future studies could consider alternative ways to operationalize social interactions and solitude. Second, our study included sleeping time in solitude episodes and it is subject to debate whether sleeping in the presence of others, such as the partner, could be counted as being in solitude. Older adults typically sleep 7–8 hr (Vincent et al., 2020). In our study, there were only 55 (2%) episodes that lasted 8 hr or shorter and 245 (10%) episodes lasted 10 hr or shorter. We take the fact that overnight solitude episodes were relatively prevalent (22%) and lasted relatively long (*M* = 16.94 hr) as indication that these episodes also included meaningful waking solitude time (besides sleeping). We thus considered the bias that might be introduced by including sleep time to be limited. Nevertheless, we acknowledge that sleeping is crucial for energy restoration and the role that sleep might play in the process of alternating time allocation should be carefully examined by future research. Third, we note that although the effect sizes of the interaction‐then‐solitude association and the solitude‐then‐interaction association were similar, the explained variances of the two associations beyond the covariates differed (see Table S1). To start with, the effect size metrics of STE is not commonly used in multilevel modelling and its properties are not well understood (Hülür et al., 2014). We also acknowledge that the discrepancy might be due to the fact that the residuals of the multilevel models were not perfectly normally distributed. We note that we replicated our findings (under the omission of random effects for duration to achieve model convergence) using an R package for robust estimation of linear mixed‐effects models: robustlmm (Koller, 2016). Although multilevel models are generally robust in dealing with skewness (Maas & Hox, 2004), it may have influenced the calculation of the STEs. Further research is needed to replicate our findings. Relatedly, we also note that the unstandardized coefficient (in terms of the increase in minutes) for the solitude‐then‐interaction model was quite small and future research should further understand the practical implications of the small increase in social interaction duration. Fourth, associations between social interactions, solitude, well‐being and fatigue could be complex, depending on various contextual and personal factors. Future studies could capture diverse information of activities and psychological experiences as well as time spent on social interactions and solitude using a multimethod approach combing self‐report and sensing technologies (Demiray et al., 2020; Sun et al., 2020). Finally, this study included a sample of community‐dwelling and retired older adults with relatively high education, income and health status. Regulatory will and capacity for time arrangement of daily activities could be influenced by older adults' own personal resources (e.g. health status, cognitive abilities, personality) as well as environmental opportunities (e.g. available social ties, cultural and societal norms, finance; Cornwell & Laumann, 2018; Luo & Chui, 2016; Pickett et al., 2020; Vagni & Cornwell, 2018; Vilhelmson et al., 2021). Thus, future studies could consider examining the role of agency and adaptation strategies in time spent on daily activities with more heterogeneous samples of older adults.

## CONCLUSION

This study used an event‐contingent design to examine alternation of time spent on social interactions and solitude in older adults. Results showed that longer‐than‐usual solitude episodes were followed by longer‐than‐usual social interactions and vice versa. Furthermore, older adults with higher trait well‐being and lower trait fatigue spent even more time in social interactions after longer solitude episodes, amplifying the solitude‐then‐interaction association. Our findings suggest that time spent on daily activities may reflect older adults' self‐regulatory processes. Whereas social interaction is a means to improve life satisfaction, solitude is also an integral and important part in older adults' daily life supporting energy recovery. Our findings could eventually be used to inform older adults on leading a socially fulfilled life and achieve personally optimal well‐being in light of shifting resource dynamics.

## AUTHOR CONTRIBUTIONS

**Minxia Luo:** Conceptualization; formal analysis; investigation; methodology; visualization; writing – original draft. **Theresa Pauly:** Conceptualization; investigation; methodology; visualization; writing – review and editing. **Christina Röcke:** Conceptualization; investigation; writing – review and editing. **Gizem Hülür:** Conceptualization; data curation; formal analysis; funding acquisition; investigation; methodology; project administration; resources; supervision; writing – review and editing.

## CONFLICT OF INTEREST

All authors declare no conflict of interest.

### OPEN RESEARCH BADGES

This article has earned Open Data and Open Materials badges. Materials and data are publicly accessible via the Open Science Framework (OSF).

## Supporting information


Tables S1‐S3

## Data Availability

The data that support the findings of this study are openly available in OSF: https://osf.io/rbgqn/.
