# Supplementary material for: Alternating time spent on social interactions and solitude in healthy older adults
Source: Br J Psychol. 2022 Aug 11;113(4):987–1008. doi: 10.1111/bjop.12586 (PMC9804578; doi:10.1111/bjop.12586)
Supplement: Supplementary file 1 — Tables S1‐S3 [file BJOP-113-987-s001.docx]

**Supplementary Materials**

Table 1.

*Effects of Prior Activity Duration on Subsequent the Other Activity Duration with Covariates.*

| Parameter | **Subsequent Solitude Duration** | | | **Subsequent Social Interaction Duration** | | |
| --- | --- | --- | --- | --- | --- | --- |
| Fixed effects | Est. | SE | STE | Est. | SE | STE |
| Intercept (γ_00_) | 7.47* | 0.64 | 1.33 | 0.69* | 0.06 | 3.85 |
| Prior activity duration (social interaction / solitude) (γ_10_) | 0.27* | 0.07 | 0.05 | 0.01* | 0.003 | 0.07 |
| Age | 0.39* | 0.13 | 0.25 | 0.01 | 0.01 | 0.07 |
| Gender | 0.84 | 1.44 | 0.06 | 0.04 | 0.10 | 0.03 |
| Health conditions | −0.02 | 0.33 | −0.01 | −0.02 | 0.02 | −0.04 |
| Marital status | 1.72 | 1.44 | 0.13 | −0.11 | 0.10 | −0.10 |
| Occasion | 0.02 | 0.01 | 0.02 | 0.004* | 0.002 | 0.05 |
| Weekend | 0.97* | 0.16 | 0.06 | 0.17* | 0.03 | 0.13 |
| Random effects |  |  |  |  |  |  |
| SD (intercept, u_0i_) | 6.70 |  |  | 0.56 |  |  |
| SD (slope, u_1i_) | 0.40 |  |  | 0.03 |  |  |
| Corr (intercept-slope) | −0.08 |  |  | 0.75 |  |  |
| SD (residual, e_ti_) | 7.14 |  |  | 1.26 |  |  |
| Pseudo R-Squared^a^ | 25.9% |  |  | 18.4% |  |  |

*Note*. Est. = estimate; SE = standard error; STE = standardized regression estimate; SD = standard deviation; Corr = correlation.

^a^ The pseudo R-squared of the models with only covariates (age, gender, health conditions, marital status, occasion, weekend) was 25.7% and 12.7%.

**p* < .05.

Table 2.

*Effects of Prior Social Interactions Duration on Subsequent Solitude Duration by Wellbeing and Fatigue with Covariates*.

|  |  |  | **Outcome: Subsequent Solitude Duration** | | | | | | | | | | |  |
| --- | --- | --- | --- | --- | --- | --- | --- | --- | --- | --- | --- | --- | --- | --- |
| Parameter | | | **Moderator: Positive Affect** | | | **Moderator:**  **Negative Affect** | | | **Moderator:**  **Life Satisfaction** | | | **Moderator:**  **Fatigue** | | |
| Fixed effects | | | Est. | SE | STE | Est. | SE | STE | Est. | SE | STE | Est. | SE | STE |
| Intercept (γ_00_) | | | 7.50* | 0.63 | 1.34 | 7.47* | 0.64 | 1.33 | 7.49* | 0.64 | 1.34 | 7.47* | 0.64 | 1.33 |
| Prior social interaction duration (γ_10_) | | | 0.27* | 0.08 | 0.05 | 0.28* | 0.07 | 0.05 | 0.27* | 0.07 | 0.05 | 0.28* | 0.07 | 0.05 |
| Moderator (γ_01_) | | | −1.56* | 0.65 | −0.23 | 0.45 | 0.71 | 0.07 | −1.42* | 0.57 | −0.19 | −0.31 | 0.80 | −0.04 |
| Prior social interaction duration ×  Moderator (γ_11_) | | | −0.10 | 0.09 | −0.02 | 0.07 | 0.08 | 0.01 | −0.10 | 0.07 | −0.02 | 0.08 | 0.07 | 0.01 |
| Age | | | 0.34* | 0.13 | 0.22 | 0.39* | 0.13 | 0.26 | 0.37* | 0.13 | 0.24 | 0.40* | 0.13 | 0.26 |
| Gender | | | 0.59 | 1.40 | 0.04 | 0.74 | 1.45 | 0.05 | 0.63 | 1.41 | 0.05 | 0.83 | 1.47 | 0.06 |
| Health conditions | | | −0.22 | 0.34 | −0.06 | −0.14 | 0.38 | −0.04 | −0.20 | 0.33 | −0.06 | 0.07 | 0.41 | 0.02 |
| Marital status | | | 1.98 | 1.41 | 0.14 | 1.64 | 1.46 | 0.12 | 2.00 | 1.41 | 0.15 | 1.73 | 1.47 | 0.13 |
| Occasion | | | 0.02 | 0.01 | 0.02 | 0.02 | 0.01 | 0.02 | 0.02 | 0.01 | 0.02 | 0.02 | 0.01 | 0.02 |
| Weekend | | | 0.96* | 0.16 | 0.06 | 0.97* | 0.16 | 0.06 | 0.97* | 0.16 | 0.06 | 0.97* | 0.16 | 0.06 |
| Random effects | | |  |  |  |  |  |  |  |  |  |  |  |  |
| SD (intercept, u_0i_) | | | 6.57 |  |  | 6.72 |  |  | 6.59 |  |  | 6.73 |  |  |
| SD (slope, u_1i_) | | | 0.44 |  |  | 0.39 |  |  | 0.40 |  |  | 0.38 |  |  |
| Corr (intercept-slope) | | | −0.20 |  |  | −0.10 |  |  | −0.21 |  |  | −0.02 |  |  |
| SD (residual, e_ti_) | | | 7.14 |  |  | 7.14 |  |  | 7.14 |  |  | 7.14 |  |  |
| Pseudo R-Squared^a^ | | | 25.9% |  |  | 25.9% |  |  | 25.9% |  |  | 25.9% |  |  |

*Note*. Est. = estimate; SE = standard error; STE = standardized regression estimate; SD = standard deviation; Corr = correlation.

^a^ The pseudo R-squared of the models with only covariates (age, gender, health conditions, marital status, occasion, weekend) was 25.7%.

**p* < .05

Table 3.

*Effects of Prior Solitude Duration on Subsequent Social Interaction Duration by Wellbeing and Fatigue with Covariates*.

|  |  |  | | **Outcome: Subsequent Social Interaction Duration** | | | | | | | | | | |  |
| --- | --- | --- | --- | --- | --- | --- | --- | --- | --- | --- | --- | --- | --- | --- | --- |
| Parameter | | | **Moderator: Positive Affect** | | | | **Moderator:**  **Negative Affect** | | | **Moderator:**  **Life Satisfaction** | | | **Moderator:**  **Fatigue** | | |
| Fixed effects | | | Est. | | SE | STE | Est. | SE | STE | Est. | SE | STE | Est. | SE | STE |
| Intercept (γ_00_) | | | 0.69* | | 0.06 | 3.85 | 0.69* | 0.06 | 3.86 | 0.69* | 0.06 | 3.85 | 0.69* | 0.06 | 3.84 |
| Prior solitude duration (γ_10_) | | | 0.01* | | 0.003 | 0.07 | 0.01* | 0.003 | 0.07 | 0.01* | 0.003 | 0.07 | 0.01* | 0.003 | 0.07 |
| Moderator (γ_01_) | | | 0.04 | | 0.06 | 0.07 | −0.10 | 0.06 | −0.19 | 0.07 | 0.06 | 0.11 | −0.13* | 0.06 | −0.23 |
| Prior solitude duration ×  Moderator (γ_11_) | | | 0.003 | | 0.003 | 0.02 | −0.002 | 0.003 | −0.01 | 0.01* | 0.003 | 0.04 | −0.01* | 0.003 | −0.03 |
| Age | | | 0.01 | | 0.01 | 0.07 | 0.01 | 0.01 | 0.06 | 0.01 | 0.01 | 0.07 | 0.01 | 0.01 | 0.08 |
| Gender | | | 0.04 | | 0.10 | 0.03 | 0.05 | 0.10 | 0.04 | 0.04 | 0.10 | 0.04 | 0.01 | 0.10 | 0.01 |
| Health conditions | | | −0.01 | | 0.02 | −0.04 | 0.01 | 0.03 | 0.02 | −0.01 | 0.02 | −0.04 | 0.01 | 0.03 | 0.03 |
| Marital status | | | −0.11 | | 0.10 | −0.10 | −0.09 | 0.10 | −0.08 | −0.11 | 0.10 | −0.10 | −0.10 | 0.10 | −0.09 |
| Occasion | | | 0.004* | | 0.002 | 0.02 | 0.004* | 0.002 | 0.02 | 0.004* | 0.002 | 0.02 | 0.004* | 0.002 | 0.02 |
| Weekend | | | 0.17* | | 0.03 | 0.06 | 0.17* | 0.03 | 0.06 | 0.17* | 0.03 | 0.06 | 0.17* | 0.03 | 0.06 |
| Random effects | | |  | |  |  |  |  |  |  |  |  |  |  |  |
| SD (intercept, u_0i_) | | | 0.56 | |  |  | 0.56 |  |  | 0.56 |  |  | 0.56 |  |  |
| SD (slope, u_1i_) | | | 0.03 | |  |  | 0.03 |  |  | 0.03 |  |  | 0.03 |  |  |
| Corr (intercept-slope) | | | 0.75 | |  |  | 0.76 |  |  | 0.75 |  |  | 0.74 |  |  |
| SD (residual, e_ti_) | | | 1.26 | |  |  | 1.26 |  |  | 1.26 |  |  | 1.26 |  |  |
| Pseudo R-Squared^a^ | | | 18.4% | |  |  | 18.4% |  |  | 18.4% |  |  | 18.4% |  |  |

*Note*. Est. = estimate; SE = standard error; STE = standardized regression estimate; SD = standard deviation; Corr = correlation.

^a^ The pseudo R-squared of the models with only covariates (age, gender, health conditions, marital status, occasion, weekend) was 12.7%.

**p* < .05.
